# A quantitative prevalence of *Escherichia coli* O157 in different food samples using real‐time qPCR method

**DOI:** 10.1002/fsn3.3055

**Published:** 2022-09-15

**Authors:** Babak Pakbin, Wolfram Manuel Brück, Thomas B. Brück, Samaneh Allahyari, Iradj Ashrafi Tamai

**Affiliations:** ^1^ Institute for Life Technologies University of Applied Sciences Western Switzerland Valais‐Wallis Sion 2 Switzerland; ^2^ Department of Chemistry, Werner Siemens Chair of Synthetic Biotechnology Technical University of Munich (TUM) München Germany; ^3^ Medical Microbiology Research Center Qazvin University of Medical Sciences Qazvin Iran; ^4^ Department of Microbiology and Immunology, Faculty of Veterinary Medicine University of Tehran Tehran Iran

**Keywords:** *E. coli* O157, food samples, qPCR, quantitative prevalence

## Abstract

*Escherichia coli* serogroup O157 is the main causative agent of several intestinal and extra‐intestinal foodborne diseases in humans through consumption of low‐dose contaminated foods such as milk, beef, and vegetables. To date, studies regarding the quantitative prevalence of *E. coli* O157 in foods are so limited. Therefore, this study aimed to evaluate the quantitative prevalence rate of *E. coli* serogroup O157 in raw milk (*n* = 144), vegetable salad (*n* = 174), and minced beef samples (*n* = 108) using the real‐time qPCR SYBR green melting curve method targeting the *rfbA* gene. First, we evaluated the method and found a sensitive and specific qPCR assay with 1 log of CFU/ml detection limit to detect *E. coli* O157 (Tm = 80.3 ± 0.1°C). About 2.77%, 10.18%, and 9.19% of raw milk, minced beef, and vegetable salad samples, respectively, were contaminated with *E. coli* O157. Minced beef and vegetable salad samples were significantly more contaminated than raw milk samples. Population average of *E. coli* O157 in raw milk, minced beef, and vegetable salad samples were 2.22 ± 0.57, 3.30 ± 0.40, and 1.65 ± 0.44 log CFU/ml or gr, respectively. Significantly higher levels of population of *E. coli* O157 were observed in minced beef samples. Minced beef can be regarded as the main food in the transmission of this foodborne pathogen. Routine quantitative rapid monitoring is strongly suggested to be carried out to prevent foodborne diseases caused by *E. coli* O157.

## Graphical abstract

Escherichia coli serogroup O157 is the main causative agent of several intestinal and extra‐intestinal foodborne diseases in human through consumption of low‐dose contaminated foods such as milk, beef and vegetables. To date, studies regarding the quantitative prevalence of E. coli O157 in foods are so limited. Therefore, the aim of this study was to evaluate the quantitative prevalence rate of E. coli serogroup O157 in raw milk, vegetable salad and minced beef samples using real time qPCR SYBR green melting curves targeting the rfbA gene. We found that Minced beef can be regarded as the main food in transmission of this foodborne pathogen. Routine quantitative rapid monitoring is strongly suggested to be carried out to prevent foodborne diseases caused by E. coli O157.

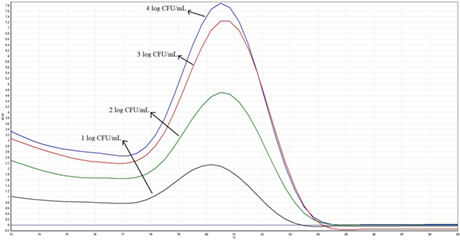

## 1 INTRODUCTION

Foodborne or waterborne disease occurs when a foodborne or waterborne pathogen is ingested with food or water and establishes itself in the human or animal host. It may also occur when a toxigenic foodborne or waterborne pathogen establishes itself in a food or water supply and produces different intestinal and extra‐intestinal toxins which are ingested (Todd, 2020). More than 250 different foodborne hazards have been recognized (Hoffmann & Scallan, 2017). Notably, most severe foodborne cases occur in the very old, the very young, pregnant, and immune compromised individuals (Braden & Tauxe, 2013). Food borne diseases are a great threat to public health and be regarded as the main concern and challenge in food industry, health, hand safety (Devleesschauwer et al., 2018). More than 76 million foodborne cases, 320,000 hospitalizations, and 5000 deaths have been reported annually in the United States (Mead et al., 1999). The World Health Organization declared that more than 1.5 million people died from the acute diarrheal diseases caused by different foodborne pathogens around the world (Pires et al., 2021). The most common foodborne bacterial pathogens are pathogenic *Escherichia coli*, *Salmonella* spp., *Campylobacter* spp., *Staphylococcus aureus*, *Clostridium perfringens*, *Listeria monocytogenes*, *Shigella* spp., and *Cronobacter sakazakii* (Gourama, 2020).

*Escherichia coli* is a rod‐shaped, Gram‐negative, facultative anaerobe, nonsporulating bacterium belonging to the *Enterobacteriaceae* family, which is part of the normal gastrointestinal microbiota of humans and animals. It is also commonly found in abundance in soil and water (Denamur et al., 2021). Different serotypes of *E. coli* have been identified and isolated from clinical, food, water, and environmental samples. *Escherichia coli* O157 has been known as the most common serotype of pathogenic *E. coli* strains identified as enterohemorrhagic and Shiga‐toxin producing bacterial foodborne pathogens (Rani et al., 2021). In 1982, the first outbreaks caused by *E. coli* O157 were reported in Michigan and Oregon states, USA. The organism was isolated from patients with abdominal cramp and severe bloody diarrhea after consumption of hamburgers in local restaurants (Lim et al., 2010; Pennington, 2010). *Escherichia coli* serotype O157: H7 is now recognized as the main causative agent of hemolytic uremic syndrome, hemorrhagic colitis, permanent organ failure, and thrombotic thrombocytopenic purpura in human of all ages (Rangel et al., 2005). *Escherichia coli* serogroup O157 accounts for more than 20% of all foodborne diseases around the world (Tack et al., 2021). More than 350 outbreaks caused by *E. coli* O157: H7 occurred between 1982 and 2006 in USA (Manning et al., 2008). More than 52% of these outbreaks linked with food consumption, 9% with consumption of contaminated water, and other ones were related with person to person and animal contacts. It is interesting to note that most of these outbreaks were associated with the consumption of contaminated unpasteurized milk, salads, and poorly cooked meat products. Consequently, it has been regarded as one of the most important foodborne bacterial pathogens (Heiman et al., 2015; Kim et al., 2020; Tack et al., 2021).

Hospitalization and fatality rates of *E. coli* O157: H7 are significantly higher than that of other *E. coli* serogroups and foodborne pathogens including *Campylobacter* and *Salmonella*. The infectious dose of this foodborne pathogen is 10–100 bacterial cells, leading to different intestinal or extra‐intestinal diseases, which might progress into the stages that threaten human life (Rani et al., 2021; Singha et al., 2022). Several strategies have previously been investigated to control and prevent the spread of *E. coli* O157 as recommended by center for disease control (CDC) (Singha et al., 2022). Quantitative prevalence evaluation of different food samples has been reported as one of the most efficient strategies to control and prevent any outbreak caused by this pathogen (Laidlaw et al., 2019). Limited studies have been conducted on quantitative prevalence of *E. coli* O157 serogroup in different food samples in Iran or other parts of the world (Pakbin et al., 2020). Therefore, the aim of this study was to investigate the quantitative prevalence of *E. coli* O157 serogroup, by using real‐time qPCR of different food samples collected from different areas of Tehran city, Iran.

## 2 MATERIALS AND METHODS

### 2.1 Collection of food samples

A total number of 426 different food samples including raw cow's milk (*n* = 144), minced beef (*n* = 108), and vegetable salad (*n* = 174) samples were purchased and collected from 36 local markets located in various areas of Tehran city, Iran, between July 2021 and February 2022. All food samples were collected and transported immediately and aseptically in sterile containers and cool boxes (4°C) with ice packs to the central microbiology laboratory for total DNA extraction and further molecular analysis.

### 2.2 Bacterial strains

In this study, *E. coli* O157 (ATCC 43888) and *E. coli* non‐O157 (ATCC BAA‐2326; ATCC 9637; ATCC 25922; ATCC 27551; ATCC 8739 and ATCC 27325) reference strains were used as controls, respectively. All strains were purchased from Pasteur Institute (Pasteur In.,). Reference strains were grown in tryptic soy broth (TSB, Merck,) and cultured at 37°C for 24 h. Ten‐fold dilution series of the reference strains (positive and negative controls) were prepared to design and develop the real‐time PCR assay in this study. Serially diluted cultures were subjected to total DNA extraction.

### 2.3 DNA extraction

Food samples preparation was performed according to the method previously described by Fukushima et al. (2007) and Liu et al. (2019) (Fukushima et al., 2007; Liu et al., 2019). Prepared food samples were subjected to total genome extraction by using the SinaClon commercial total DNA extraction kit (SinaClon Co.,) according to the manufacturer’s instructions. The quality and quantity of the extracted total genomes were evaluated using the NanoDrop‐1000 spectrophotometer (ThermoFisher,). Concentrations of the all extracted DNA were adjusted to 50 ng. μL^−1^ before real‐time PCR assay. DNA templates were kept at −20°C until the further analysis.

### 2.4 Real‐time PCR assay

Real‐time PCR was carried out with an Ampliqon 2X SYBR Green Master Mix (Ampliqon,) on a Rotor‐Gene Q 6000 real‐time PCR machine (Qiagen Corbett,). Serogroup‐specific primers of *rfbA* gene, presenting in *E. coli* O157, used in this study for PCR including forward (5'‐CGGACATCCATGTGATATGG‐3') and reverse (5'‐TTGCCTATGTACAGCTAATCC‐3') primers (Burrus et al., 2017). Real‐time PCR tubes contained 10 μl of the 2X real‐time PCR master mix, 1 μl of each primer (20 μM), 1 μl of the DNA template (50 ng. μL^−1^), and sterilized nuclease free water up to a final reaction volume of 20 μl. The amplification procedure of real‐time PCR was performed using the following conditions: initial denaturation step at 94°C for 5 min, followed by 40 cycles of denaturation at 94°C for 40 s, annealing at 60°C for 40 s, and extension at 72°C for 30 s, followed by a melting step with the increasing temperature from 70 to 95°C with the raising temperature rate of 0.2°C/s. The size of amplicons produced by *rfbA* primers was 242 bp. The analysis of amplification and melting curves was carried out using the Rotor‐Gene 6000 software version 2.3.5 (Qiagen Corbett,).

### 2.5 Statistical analysis

One‐way analysis of variance (ANOVA) and Chi‐square tests were used to evaluate the significant differences (*p* < .05) between the contamination rates and the average microbial populations, respectively, using the SPSS software version 23.0.1. All experimental and statistical measurements were performed in triplicate.

## 3 RESULTS

**FIGURE 1:**
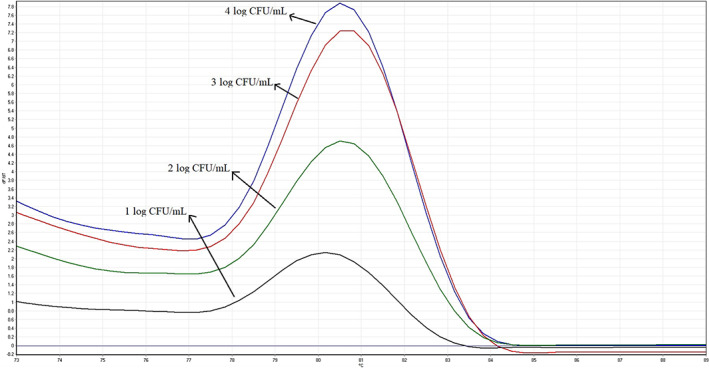
Melting curves of real‐time PCR using a 10‐fold dilution series of *E. coli* serogroup O157

**FIGURE 2:**
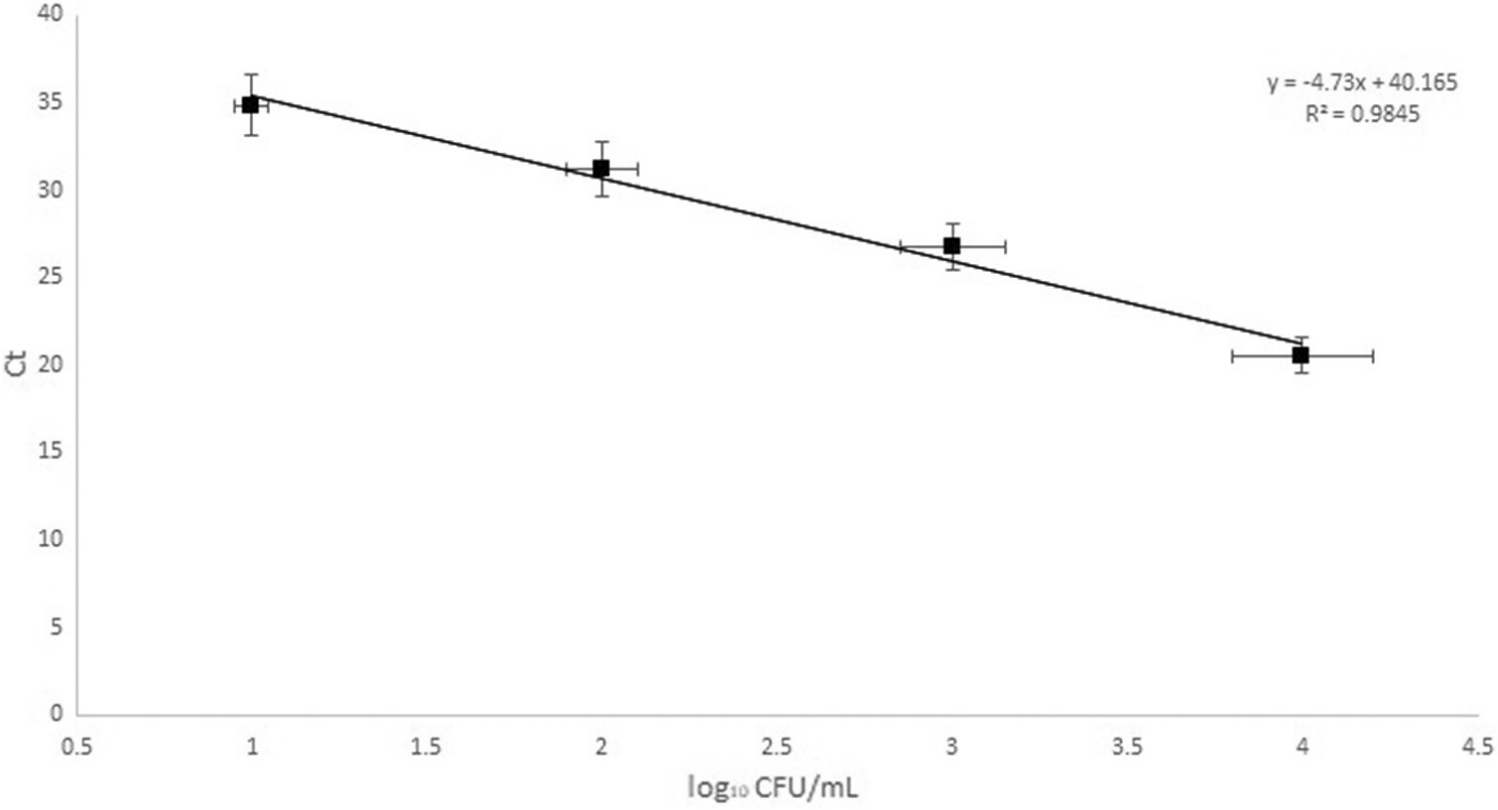
Standard curve of quantitative PCR using a 10‐fold dilution series of *E. coli* serogroup O157

In this study, we have used qPCR to investigate the quantitative prevalence of *E. coli* serogroup O157 in different food samples. First of all, we developed and optimized the qPCR method to quantify the *E. coli* O157 counts. We detected the *rfbA* gene in *E. coli* O157 strain for identification of this foodborne pathogen through real‐time PCR assay at the present study. Figure 1 represents the melting curves of PCR amplicons of decimally diluted pure broth culture of *E. coli* O157 DNA templates to determine the limit of detection of the method. The average melting temperature (T_m_) of *rfbA* amplicon generated in real‐time PCR was 80.3 ± 0.1 °C. No non‐O157 *E. coli* strains was identified by using the developed method. The limit of detection for *rfbA* primer set using DNA extracted from serially diluted pure broth culture of *E. coli* serogroup O157 was found to be 10 CFU/ml (1 log of CFU/ml) with the associated C_t_ value. After plotting the standard curve, the amplification efficiency was calculated 97.8%. Standard curve of the real‐time PCR for quantification of *E. coli* O157 count generated by plotting the C_t_ value of reactions against the *E. coli* O157 log of the CFU/ml was demonstrated in Figure 2. After recovering *E. coli* serogroup O157, the negative and positive results of detection were confirmed by the developed real‐time PCR method. As shown in Figure 2, the detection limit of the reference strain also was 1 log. The regression value of the standard curve equation (*R* ^2^ = 0.98) in Figure 2 showed a significant relationship between the C_t_ values and bacterial counts in the developed qPCR method. We found real‐time qPCR method, targeting the *rfbA* gene, a sensitive and specific assay to investigate the quantitative prevalence of *E. coli* O157 in food samples.

**FIGURE 3:**
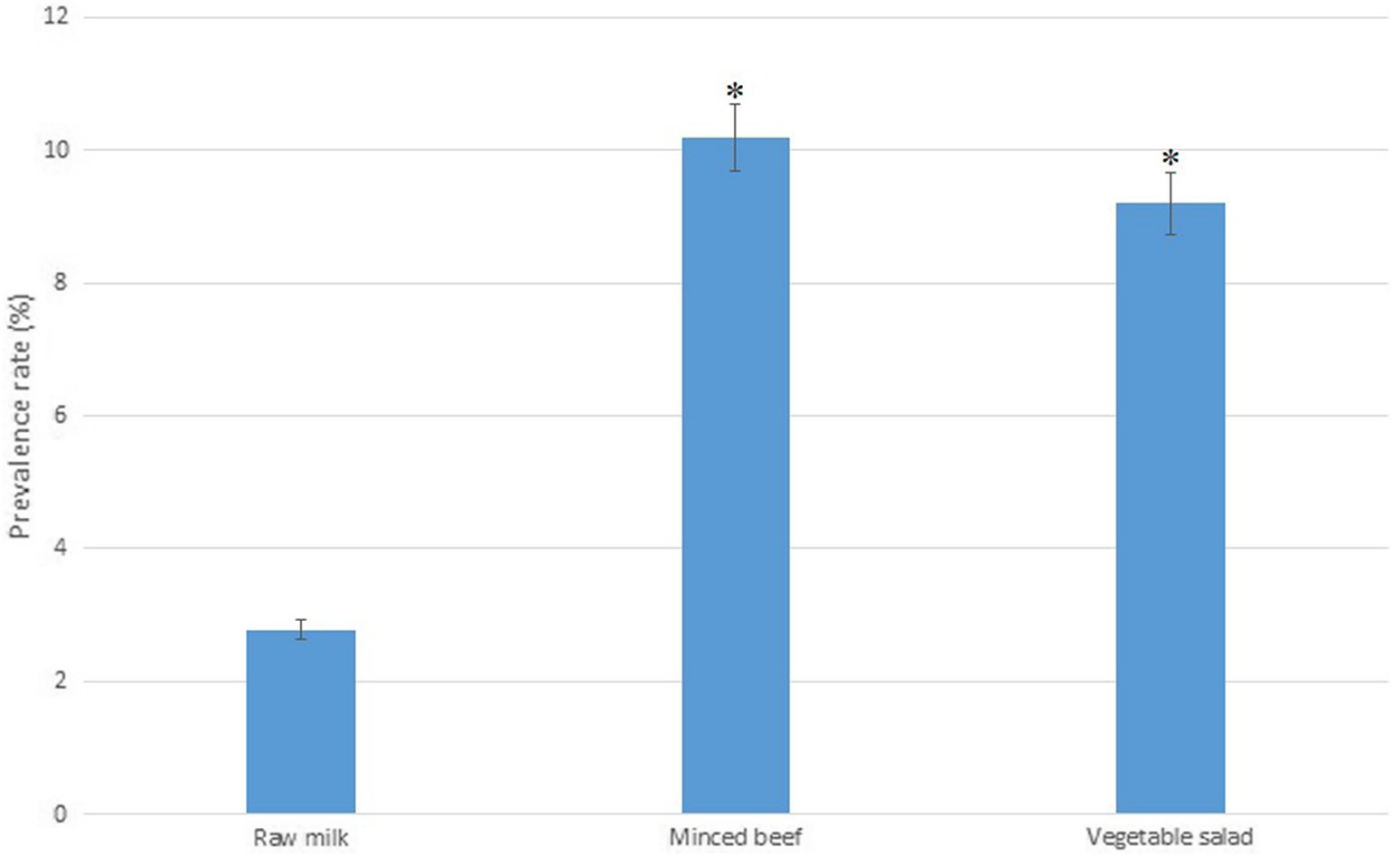
Prevalence rate of *E. coli* serogroup O157 in raw milk, minced beef and vegetable salad samples. * indicates significant differences (*p* < .05)

The prevalence rate of *E. coli* O157 in different food samples including minced beef, vegetable salad, and raw milk was evaluated by using a sensitive and specific real‐time qPCR method which was developed in this study. Figure 3 illustrates the prevalence of *E. coli* serogroup O157 in raw milk, minced beef, and vegetable salad samples. About 2.77% (4 out of 144), 10.18% (11 out of 108), and 9.19% (16 out of 174) of raw milk, minced beef, and vegetable salad samples, respectively, were contaminated with *E. coli* serogroup O157 in this study. In total, *E. coli* O157 was detected in 7.27% (31 out of 426) of food samples. *Escherichia coli* O157 was also detected significantly (*p* < .05) in higher levels in minced beef and vegetable salad samples than that in raw milk samples. Populations of *E. coli* O157 serogroup in different *E. coli* O157‐positive food samples are shown in Table 1. As it can be seen in Table 1, average population of *E. coli* O157 in raw milk, minced beef, and vegetable salad samples were observed 2.22 ± 0.57, 3.30 ± 0.40, and 1.65 ± 0.44 log CFU/ml or gr, respectively. The average population of *E. coli* O157 in minced beef samples was significantly (*p* < .05) higher than that observed in raw milk and vegetable salad samples. Also, the concentrations of *E. coli* O157 varied considerably among different food samples, which ranged from 1.5 to 2.9 log CFU/ml, from 2.7 to 4.0 log CFU/mg, and from 1.1 to 3.0 log CFU/mg in raw milk, minced beef, and vegetable salad samples, respectively.

**TABLE 1:** Population of *E. coli* O157 in different food samples

Sample number	Food samples	Ct	Population (log CFU/ml or gr)	Average population (log CFU/ml or gr)^a^
4	Raw milk	33.07	1.5	2.22 ± 0.57
	Raw milk	29.28	2.3	
	Raw milk	26.44	2.9	
	Raw milk	29.75	2.2	
11	Minced beef	23.61	3.5	3.30 ± 0.40
	Minced beef	27.39	2.7	
	Minced beef	23.61	3.5	
	Minced beef	25.97	3.0	
	Minced beef	24.08	3.4	
	Minced beef	22.66	3.7	
	Minced beef	25.97	3.0	
	Minced beef	25.02	3.2	
	Minced beef	23.13	3.6	
	Minced beef	21.24	4.0	
	Minced beef	26.92	2.8	
16	Vegetable salad	32.59	1.6	1.65 ± 0.44
	Vegetable salad	33.07	1.5	
	Vegetable salad	32.59	1.6	
	Vegetable salad	29.75	2.2	
	Vegetable salad	33.54	1.4	
	Vegetable salad	31.65	1.8	
	Vegetable salad	25.97	3.0	
	Vegetable salad	32.59	1.6	
	Vegetable salad	34.01	1.3	
	Vegetable salad	32.59	1.6	
	Vegetable salad	31.65	1.8	
	Vegetable salad	32.12	1.7	
	Vegetable salad	34.48	1.2	
	Vegetable salad	34.96	1.1	
	Vegetable salad	32.59	1.6	
	Vegetable salad	33.07	1.5	

^a^ Significant differences (*p* < .05) among the population averages.

## 4 DISCUSSION

*Escherichia coli* O157 is the most common serogroup member of pathogenic *E. coli* strains identified differently as Shiga‐toxin producing, verotoxin producing, and enterohemorrhagic bacterial pathogen. *Escherichia coli* O157 is recognized as particularly as virulent foodborne bacterial pathogen which only requires a small number of bacterial cells to cause disease in the host (Pakbin et al., 2021). Also, low levels of contamination have been detected in the infected foods, which were consumed and caused to several food poisoning cases (Franz et al., 2019). This foodborne pathogen caused different intestinal and extra‐intestinal illnesses such as acute bloody diarrhea and HUS in human (Kim et al., 2020). Cattle have been recognized mainly as the most important reservoir of *E. coli* serogroup O157 causing zoonotic diseases through consumption of various foods contaminated with bovine feces (e.g., vegetables contaminated with animal‐based agricultural fertilizers), undercooked meat, and unpasteurized dairy products in humans (Gutema et al., 2021). However, this bacterial pathogen has also been isolated from naturally acquired infections from the consumption of other animal species such as sheep, deer, goat, moose, chicken, turkey, among others. Thus, *E. coli* O157 has been considered as one of the main challenges and major concerns in public health and food safety in developing and developed countries around the world (Abreham et al., 2019).

Gold standard methods currently rely on pre‐enrichment and culture of *E. coli* serogroup O157 from different contaminated foods. These methods are laborious and time‐consuming (Pang et al., 2018). Molecular assays such as polymerase chain reaction (PCR)‐based methods are considerably more sensitive. However, these methods are relatively expensive and require instrumentation. qPCR is a specific molecular PCR‐based assay commonly used to quantify different bacterial strains (DNA) in food samples (Wei et al., 2018). qPCR method provides faster, safer, cheaper, and more practical strategy than conventional methods for diagnosis and quantification of different foodborne pathogens in food sample, and in the recent decades, this method is strongly appreciated by clinicians and would aid their suitable treatment of their patients with foodborne diseases (Bustin, 2010; Kubista et al., 2006; Yang & Rothman, 2004). Also, the simplicity and high‐throughput adaptability of the method would enable rapid diagnostics of spoiled food improving food safety over the entire food production and processing chain in developing countries. Moreover, in a clinical setting, it would enable a rapid treatment response that does safe life particularly when patients have to travel from remote areas to seek treatment and where on presentation toxic symptoms and systemic infection are often in an advanced state (Chen et al., 2021). However, the main limitation of this method is its inability to differentiate live from dead cell DNA (Hu et al., 2018). qPCR assays were used to detect and quantify *E. coli* serogroup O157 strains in different clinical, environmental, and food samples (Kim & Oh, 2021). In this study, we also used real‐time qPCR SYBR green melting curves, targeting the *rfbA* gene, to determine the prevalence rate and quantify *E. coli* O157 in food samples. We found this method very specific and sensitive for the detection of *E. coli* O157 in food samples (detection limit of the developed method was observed 1 log of CFU/ml for pure broth culture of *E. coli* O157). Azinheiro et al. (2020) developed new assay based on SYBR green melting curve qPCR method to detect three foodborne pathogens including *E. coli* O157 and other bacterial pathogens simultaneously in spiked out infant formula samples. They found that this method is specific and sensitive and has the detection limit of <1 CFU/25 gr to detect *E. coli* O157 in the contaminated infant formula samples (Azinheiro et al., 2020). Kim et al. (2020) designed and optimized qPCR method to detect *E. coli* O157 and *Salmonella typhimurium* in cabbage and iceberg without any pre‐enrichment step. They demonstrated that this method was able to detect 7 CFU of *E. coli* O157 in 25 g of iceberg and cabbage samples (Kim & Oh, 2021). Several other researchers also declared that qPCR SYBR green melting curve analysis method is sufficiently sensitive and specific with an excellent limit of detection to detect *E. coli* O157 in food samples in single and multiplex reactions (Barbau‐Piednoir et al., 2018).

In this study, 2.77%, 10.18%, and 9.19% of raw milk, minced beef, and vegetable salad samples, respectively, were contaminated with *E. coli* O157. We found that the prevalence rates of *E. coli* O157 strains in minced beef and vegetable salad samples were significantly higher than that in raw milk samples. El‐Atty and Meshref (2008) reported lower prevalence rates of *E. coli* O157 in milk and dairy products (2% and 4%) in Egypt than that we detected in this study (El‐Atty & Meshref, 2008). Abong'o et al. (2008) in South Africa found *E. coli* O157 in 10.3% (4 out of 39) of vegetable samples, which was similar to the contamination rate detected in this study (Abong'o et al., 2008). In another study from Egypt, Sallam et al. (2013) demonstrated that the contamination rates of *E. coli* O157 in minced beef, beef burger, and fresh beef samples were 26.7% (8 out of 30), 10% (3 out of 30), and 3.7% (1 out of 27), respectively. They reported that 13.8% of all beef samples were contaminated with this foodborne pathogen, which was significantly higher than that we reported in this study (Sallam et al., 2013). Recently, Ahmad et al. (2021) investigated the prevalence rate of *E. coli* O157 in raw milk samples from farms in Pakistan, and they reported that 6% (6 out 0f 100) of unpasteurized milk samples were contaminated with *E. coli* O157: H7, which was significantly higher than that we observed in this study. Due to the various hygienic measures throughout the milk production processes such as implementation of different hygiene protocols, screening of this pathogen during the storage time, hygiene status of the equipment and training of milk production workers, different prevalence rates of *E. coli* O157 have been reported around the world (Ahmad et al., 2021). We also found that the average population of *E. coli* O157 in minced beef samples was significantly higher than that we observed in raw milk and vegetable salad samples. Consequently, minced beef can be considered as the most important food transmitting *E. coli* serogroup O157 into the consumers. Furukawa et al. (2018) found that consumption of minced beef contaminated with *E. coli* O157 caused a gastroenteritis outbreak in 2016 in Kanagawa, Japan (Furukawa et al., 2018). Cooking sufficient and safe and hygienic food handling and distribution procedures are strongly suggested to be used to prevent infection and gastroenteritis outbreaks caused by *E. coli* O157 via consumption of contaminated foods, especially minced meat products (Burrus et al., 2017; Rangel et al., 2005; Rani et al., 2021).

## 5 CONCLUSION

In conclusion, we showed that real‐time qPCR SYBR green melting curve analysis is a sensitive and specific assay with a low detection limit for the detection and identification of *E. coli* serogroup O157 in food samples. Also, we found that minced beef and vegetable salad samples were significantly more contaminated with *E. coil* O157 than raw milk samples. The population average of *E. coli* serogroup O157 was observed in significantly higher levels in minced beef samples than that in raw milk and vegetable salad samples. Sufficient thermal treatments, hygienic practices, and routine quantitative rapid monitoring are strongly recommended to be implemented to prevent foodborne diseases and outbreaks caused by *E. coli* O157 through consumption of contaminated foods.

## CONFLICT OF INTEREST

All the authors have declared no conflict of interest.

## Data Availability

We confirm that all data and findings of this study are available within the article.
